# Which clinical and biochemical predictors should be used to screen for diabetes in patients with serious mental illness receiving antipsychotic medication? A large observational study

**DOI:** 10.1371/journal.pone.0210674

**Published:** 2019-09-12

**Authors:** Alex J. Mitchell, Davy Vancampfort, Peter Manu, Christoph U. Correll, Martien Wampers, Ruud van Winkel, Weiping Yu, Marc De Hert

**Affiliations:** 1 University of Leicester, Leicester, England, United Kingdom; 2 University Psychiatric Center, Catholic University Leuven, Kortenberg, Belgium; 3 University Psychiatric Center, Kortenberg, Belgium; 4 School of Mental Health and Neuroscience (EURON), University Medical Center, Maastricht, The Netherlands; 5 Zucker Hillside Hospital, Glen Oaks, New York, United States; 6 Hofstra North Shore–LIJ School of Medicine, Hempstead, New York, United States; FIDMAG Research Foundation, SPAIN

## Abstract

**Objective:**

We aimed to investigate which clinical and metabolic tests offer optimal accuracy and acceptability to help diagnose diabetes among a large sample of people with serious mental illness in receipt of antipsychotic medication.

**Methods:**

A prospective observational study design of biochemical and clinical factors was used. Biochemical measures were fasting glucose, insulin and lipids, oral glucose tolerance testing (OGTT), hemoglobin A1c, and insulin resistance assessed with the homeostatic model (HOMA-IR) were determined in a consecutive cohort of 798 adult psychiatric inpatients receiving antipsychotics. Clinical variables were gender, age, global assessment of functioning (GAF), mental health clinicians’ global impression (CGI), duration of severe mental illness, height, weight, BMI and waist/hip ratio. In addition, we calculated the risk using combined clinical predictors using the Leicester Practice Risk Score (LPRS) and the Topics Diabetes Risk Score (TDRS). Diabetes was defined by older criteria (impaired fasting glucose (IFG) or OGTT) as well as2010 criteria (IFG or OGTT or Glycated haemoglobin (HBA1c)) at conventional cut-offs.

**Results:**

Using the older criteria, 7.8% had diabetes (men: 6.3%; women: 10.3%). Using the new criteria, 10.2% had diabetes (men: 8.2%, women: 13.2%), representing a 30.7% increase (p = 0.02) in the prevalence of diabetes. Regarding biochemical predictors, conventional OGTT, IFG, and HbA1c thresholds used to identify newly defined diabetes missed 25%, 50% and 75% of people with diabetes, respectively. The conventional HBA1c cut-point of ≥6.5% (48 mmol/mol) missed 7 of 10 newly defined cases of diabetes while a cut-point of ≥5.7% improved sensitivity from 44.4% to up to 85%. Specific algorithm approaches offered reasonable accuracy. Unfortunately no single clinical factor was able to accurately rule-in a diagnosis of diabetes. Three clinical factors were able to rule-out diabetes with good accuracy namely: BMI, waist/hip ratio and height. A BMI < 30 had a 92% negative predictive value in ruling-out diabetes. Of those not diabetic, 20% had a BMI ≥ 30. However, for complete diagnosis a specific biochemical protocol is still necessary.

**Conclusions:**

Patients with SMI maintained on antipsychotic medication cannot be reliably screened for diabetes using clinical variables alone. Accurate assessment requires a two-step algorithm consisting of HBA1c ≥5.7% followed by both FG and OGTT which does not require all patients to have OGTT and FG.

## Introduction

A rising population rate of overweight and obesity has contributed to a global diabetes epidemic, with harmful effects on mortality and morbidity worldwide [1]. Diabetes afflicts an estimated 382 million people and by 2035 this will rise to 592 million but many remain undiagnosed [2]. Diabetes implies a significant abnormality in glucose homeostasis with persistent hyperglycaemia. However, the exact definition of diabetes offered by expert committees has varied over time. In 1997, diabetes was defined by either an impaired fasting glucose (IFG) >125 mg/dL (≥7.0 mol/L) or a two-hour oral glucose tolerance test (OGTT) >199 mg/dL (>11 mmol/L). In 2010, Glycated haemoglobin (HBA1c) had been added to the qualifying criteria, such that diabetes mellitus can now be defined by one of three of the following: elevated fasting glucose (?7.0 mol/L), 2-hour OGTT (>11 mmol/L) or HBA1c (>6.4%; 46 mmol/mol)[3].Usually the abnormal test is repeated unless there is “unequivocal hyperglycemia” [3,4]. The addition of HbA1c reflects its contribution as an independent risk of morbidity and mortality [5]. Moreover, HBA1c is convenient, as it can be measured at any time of the day without fasting. However, it is unclear whether HbA1c is as good as blood glucose for predicting diabetes complications, such as retinopathy. Certainly, higher HBA1c is a risk for future diabetes. In a systematic review of 16 cohort studies with a follow-up interval averaging 5.6 years (range: 2.8–12 years), those with an HBA1c between 6.0 to 6.5% (42 mmol/mol—48 mmol/mol) had a 5-year risk of diabetes of 25% to 50% [6]. HBA1c has a modest-to-strong correlation with IFG and OGTT. For example, up to half of people with diabetes would not be diagnosed using HbA1c, and half of those diagnosed using HbA1c would not currently be diagnosed using IFG [7, 8]. The new definition of diabetes, which incorporates HBA1c, has increased the prevalence of diabetes in all populations,[9] but has never previously been studied in patients with severe mental illness (SMI), or those maintained on antipsychotic medications.

Observational studies have reported a clear association between patients with SMI and diabetes [10]. This is concerning as diabetes is associated with a reduced quality of life and increased mortality in people with SMI [11].The risk appears particularly severe in those maintained on antipsychotics, notably most second-generation antipsychotics [12, 13]. The risk conferred by the illness and/or antipsychotics extends to other components of the metabolic syndrome. For example, De Hert et al (2007) found that 27.8% of those started on second-generation antipsychotics had new (incident) metabolic syndrome (MetS) within three years, compared to 9.8% of those treated with first-generation antipsychotic agents [14]. In studies that have assessed metabolic abnormalities in drug-naïve, first-episode patients, some have found impaired glucose tolerance or insulin resistance but others found no appreciable effect and a recent meta-analysis found only modest abnormalities in metabolic syndrome or metabolic risk factors in drug-naïve patients (exceptions may be smoking, fitness and diet)[15]. Therefore, it is assumed that antipsychotics indirectly worsen glucose regulation by promoting obesity or directly by affecting glucose regulation through insulin resistance, [16] decreased secretion of glucose-dependent insulinotropic polypeptide (GIP), increased glucagon secretion, or by impairing beta cell function [17]. In addition, antipsychotics also contribute to dyslipidemia [18].

Despite these concerns about metabolic abnormalities in patients treated with antipsychotics, only a few studies examined screening procedures for diabetes/prediabetes in this population. In a modest sample of 100 patients, De Hert et al (2006) noted that a monitoring protocol based only on fasting glucose would detect only 63.6% of patients with glucose abnormalities. They suggested combining fasting glucose with fasting insulin [19]. This sample was later expanded to 415 patients and testing procedures were re-examined [20]. Against an OGTT definition of diabetes, IFG had a sensitivity of 46.2%, but a two-step procedure of impaired fasting glucose >100mg/dl and then an OGTT only for patients positive in step 1 gave a sensitivity of 96.2%, yet both retained 100% specificity. Manu et al (2012) used the new 2010 American Diabetes Association diagnostic criteria for prediabetes and found 37% of patients treated with antipsychotics met criteria[21].Among patients with prediabetes, HBA1c (5.7–6.4 mmol/mol) was the sole defining abnormality in 41%. Agarwal (2012) found that 48% of patients with schizophrenia had high HBA1c levels ≥ 5.7[22]. Only one study, however, has investigated HBA1c in diagnosing diabetes. Hanssens et al (2006) reported a limited value of HBA1c in diagnosing diabetes in those taking antipsychotics due to low sensitivity [23]. Since that time, the definition of diabetes has been updated and research is required to identify the role of HBA1c in diagnosing diabetes. Therefore we aimed to 1. fully examine the accuracy and clinical utility of HBA1c and other markers of glucose regulation in the diagnosis of diabetes in patients taking antipsychotics and 2. to develop an algorithm for clinicians to use in clinical practice to detect diabetes in mental health populations.

Whilst the addition of non-fasting HBA1c simplifies the biochemical diagnosis of diabetes in many non-specialist settings the diagnosis of diabetes remains a challenge due to the inconvenience of biochemical testing. Necessity for biochemical tests reduces the acceptability and uptake of testing for many clinicians and patients. The European evidence based guidelines for the prevention of type 2 diabetes[24] and the International Diabetes Federation[25] have recommend the use of simple risk scoring systems to identify people at high risk of future diabetes. However, these still rely on conventional testing. Recently, several groups have developed and tested the accuracy of clinical variables to diagnose diabetes (and to a lesser extent pre-diabetes) without recourse to blood tests. These could be valuable in clinical practice if they were sufficiently accurate. Usually these clinical variables have been combined in clinical prediction algorithms or risk models [26, 27, 28, 29, 30, 31].Abbasi et al (2012) reviewed 12 such models (an additional 13 required biochemical testing)[27]. Collins et al (2011) reviewed 43 risk prediction models of which 17 were purely clinical [24]. Noble et al (2012) evaluated 94 risk prediction models and identified 7 as being the most promising for adaptation and use in routine clinical practice [26]. Risk models vary from simple to complex. The majority have been validated in North American or European study populations. It has been suggested that simple models, derived from clinical history alone could be useful in clinical practice and could reduce the cost and inconvenience of screening. For example, the Diabetes Risk Calculator derived from the National Health and Nutrition Examination Survey (NHANES) III [32] includes questions about patient age, waist circumference, history of gestational diabetes, height, race/ethnicity, hypertension, family history, and exercise. Other models include the Atherosclerosis Risk in Communities (ARIC) risk calculator, the Australian Diabetes Risk Assessment Tool (AusDrisk), the Cambridge Risk Score, FINDRISC and CANRISK (see Table 1). Even clinical models vary in complexity of risk factors and scoring. Two particularly simple models may be suitable for application in mental health settings. These are the Leicester Practice Risk Score for Diabetes (LPRS) 33 and the Topics Diabetes Risk Score (TDRS) [34]. The accuracy of these clinical models is summarized in Table 1. One challenge of these models is to yield high clinical utility in the face of a relatively low prevalence of diabetes, typically 3–6% in the general population. The value of such tools is that they can potentially be used as a simpler form of screening which might increase the uptake of screening and ultimately reduce the incidence or complications of type 2 diabetes [35, 36, 37].

**Table 1:** Summary diabetic risk clinical models from general population studies.

Study	Country	Model Description	Sensitivity	Specificity	Area under ROC	Sample Size	Youden
**Prospective (incident cases)**							
Aekplakorn et al (2006) [65]	Thailand	Thai Risk: Age, sex, BMI, abdominal obesity (waist circumference), hypertension, family history of diabetes	77	61.9	0.75	2420	0.389
Balkau et al (2008) [66]	France	Men: waist circumference, smoking status, hypertension Women: waist circumference, family history of diabetes, hypertension.	50	74	66	3817	0.24
Gao et al (2009) [67]	Mauritius	Qingdao Score:Age, sex, BMI, waist circumference, family history of diabetes	74.5	48.5	0.63	3094	0.23
Heianza et al (2013) [34]	Japan	TDRS: age, sex, family history of diabetes, current smoking habit, BMI, and hypertension	72.7	68.1	0.77	33,335	0.408
Kahn et al (2009) [68]	USA	Diabetes family history, hypertension, ethnicity, age, smoking status, waist circumference, height, resting pulse, weight	69	64	0.71	3142	0.33
Lindström et al (2003) [69]	Finland	FINDRISC: Age, BMI, waist circumference, use of blood pressure medication, history of high blood glucose, physical activity, daily consumption of vegetables	78	77	0.85	4,435	0.55
Robinson et al (2011) [70]	Canada	CANRISK: Age, BMI, waist circumference, use of blood pressure medication, history of high blood glucose, physical activity, daily consumption of vegetables	70	67	0.75	1676	0.37
Schulze et al (2007) [71]	Germany	German Diabetes Risk Score: Waist circumference, height, age, hypertension, diet, alcohol consumption, physical activity, former or current smoker	83	68	0.83	25,167	0.51
**Cross-sectional (Prevalent Cases)**							
Al-Lawati et al (2007) [72]	Oman	Oman Diabetes Risk Score: Age, waist circumference, BMI, family history of diabetes, hypertension	78.6	73.4	0.83	4881	0.52
Baan et al (1999) [73]	The Netherlands	Rotterdam Score: Age, sex, use of antihypertensive medication, obesity (BMI ≥ 30)	78	55	0.7	2364	0.33
Bang et al (2009) [74]	USA	Patient Self-Assessment Score: Age, sex, family history of diabetes, history of hypertension, obesity (BMI or waist circumference), physical activity	82	63	0.79	5258	0.45
Gao et al (2010) [75]	China	Chinese Risk Score: Age, waist circumference, family history of diabetes	89	27	0.64	6322	0.16
Glümer et al (2004) [76]	Denmark	Danish Risk Score: Age, BMI, sex, known hypertension, physical activity, family history of diabetes	76	72	0.81	6,784	0.48
Gray et al (2010) [Addition cohort] [33]	UK	LPRS: age, ethnicity, sex, first degree family history of diabetes, hypertension, waist circumference and BMI	81.1	41	0.69	6390	0.221
Gray et al (2010) [Validation cohort] [33]	UK	LPRS: age, ethnicity, sex, first degree family history of diabetes, hypertension, waist circumference and BMI	91.5	32.4	0.72	3171	0.239
Pires de Sousa et al (2009)[77]	Brazil	Brazilian Simple Prediction Model: Age, BMI, hypertension	76	67	0.77	1224	0.43
Ramachandran et al (2005)[78]	India	Age, family history of diabetes, BMI, waist circumference, physical activity	76	59	0.73	10,003	0.35

Footer: Leicester Practice Risk Score (LPRS); Topics Diabetes Risk Score (TDRS); Finish Risk Score (FINDRISC), Canadian Risk Score (CANRISK)

## Methods

### Setting

In an observational study between November 2003 and July 2007, psychiatric patients were asked by their treating psychiatrist to agree to a standardized battery of tests to identify MetS and insulin resistance. All subjects gave written informed consent and the study was approved by the University Psychiatric Center’s Ethics Committee KU Leuven campus, Kortenberg.

### Clinical and laboratory measurements

The procedure included measurements of height, weight, body mass index (BMI), waist circumference, arterial blood pressure, fasting blood glucose, insulin and lipids, Glycated hemoglobin (A1c), and a 2-hour oral glucose tolerance test (OGTT) after the ingestion of 75g of glucose. As described previously, all tests were performed in the same laboratory and using the same robust methods throughout the study period [13]. Fasting glucose, 2-hour postprandial glucose during OGGT and HBA1c data were used to define diabetes mellitus according to new and old (conventional) criteria. The older criteria were either a fasting glucose >125 mg/dl or a 2-hour postprandial glucose >199 mg/dL. The new criteria are a fasting glucose >125 mg/dL or a 2-hour postprandial glucose >199 mg/dLor an HBA1c ≥6.5% (48 mmol/mol); any of which should be repeated unless there is unequivocal hyperglycaemia. In addition, in a patient with classical symptoms of hyperglycaemia a random glucose >199 mg/dL is an acceptable test. The fasting glucose and insulin data were used for the homeostatic model assessment of insulin resistance (HOMA-IR) [38]. The weight and height were used to calculate the body mass index (BMI). The waist circumference, arterial blood pressure, and fasting glucose, triglycerides and high-density lipoprotein (HDL) cholesterol levels were also measured.

Psychiatric diagnoses were established according to DSM-IV by experienced psychiatrists who were qualified and familiar with the management of psychiatric patients taking antipsychotics. They were blinded to the study aims and affiliated with the University Psychiatric Center and responsible for the patient’s treatment. The treating psychiatrists assessed the severity of symptoms and rated them using the Global Assessment of Function (GAF) from 0 (worst) to 100 (best)[39] and the Clinical Global Impressions Severity (CGI-S) Scale from 1 (normal) to 7 (extremely ill)[40].

### Diabetes modelling

Of the many clinical models available we chose to examine two popular models: the Leicester Practice Risk Score for Diabetes (LPRS) [30] and the Topics Diabetes Risk Score (TDRS) [31]. The LPRS encompasses the following risk factors: age, ethnicity, gender, first degree family history of diabetes, hypertension, waist circumference and BMI and has been validated in an ethnically diverse European population (sensitivity 91.5%, specificity 32.4, area under the curve 0.72). The TDRS includes age, sex, family history of diabetes, current smoking habit, BMI, and hypertension and has been validated in a Japanese sample and is currently the largest study of its type (sensitivity 72.7%, specificity 68.1%, area under the curve 0.77). In addition to these combination models, we examined the diagnostic accuracy of each individual clinical risk factor.

### Inclusion and exclusion criteria

We included psychiatric patients without diabetes at baseline consecutively admitted to a single institution in Belgium. We excluded patient unable or unwilling to consent and those not taking antipsychotic medication. Thus the study cohort comprised 820 patients but 22 were excluded as they were not treated with antipsychotic drugs leaving 798 eligible patients. This provided sufficient power to analyse at least 8 predictor variables. Patients were tested and found to be free of diabetes prior to starting antipsychotics; therefore, the diabetes cases are incident cases. Altogether, 49.1% were taking antipsychotics <3 months, 3.9% 3–6 months, 5.9% 6–9 months, and 41.1% more >1 year. The proportions of patients treated with the same antipsychotic drug for more than 3 months were 81.4% for those receiving first-generation drugs, 76.0% for clozapine, 56.6% for amisulpride, 46.2% for risperidone, 46.2% for olanzapine, 38.7% for quetiapine and 12.2% for aripiprazole.

### Statistical analyses

A receiver operator characteristic (ROC) curve analysis was conducted equally weighted for false positives and false negatives tested the best single markers for ruling-in (case-finding) or ruling out (screening). We also examined sensitivity, specificity, positive predictive value (PPV), negative predictive value (NPV), likelihood ratios and clinical utility index (CUI) and relevant confidence intervals for all tests (all available from www.clinicalutlity.co.uk). In addition, we used ROC curve to calculate the optimal cut-off points for each test. For overall accuracy we used fraction correct (also known as overall accuracy = true positives plus true negatives / all cases). In order to calculate clinical utility, we used the clinical utility index. The CUI allows calculation of qualitative as well as quantitative value of a test [41, 42]. The clinical utility index takes into account both discriminatory ability and occurrence for case-finding (CUI+) and screening (CUI-) such that the positive utility index (CUI+) = sensitivity x positive predictive value and the negative utility index (CUI-) = specificity x negative predictive value. Further details are available here: www.clinicalutility.co.uk There commended qualitative grades of diagnostic accuracy were applied according to previous publications [43]. Namely the grades of the clinical utility index were > = 0.81: excellent, > = 0.64: good and > = 0.49: fair > = 0.36: poor; <0.36: very poor. Finally, algorithm approaches were investigated. Algorithm approaches attempt to improve upon acceptability / test burden. They usually start with a simple test, acceptability to the population as a whole (such as the HBA1c) and then advise a fasting test, or OGTT only if needed.

## Results

### Demographic and psychiatric characteristics

The total sample comprised 798 patients taking antipsychotic medications with a mean age of 37.7years. 61.1% were male and the most common diagnosis was schizophrenia (67.2%). 62% were smokers. Full details of demographic and psychiatric characteristics are presented in Table 2.

**Table 2:** Demographic and psychiatric characteristics.

	Total Sample (n = 798)	New Definition of Diabetes (n = 80)	No Diabetes(n = 718)
Age (years)	37.7	47.0	36.7
Male Gender	61.1%	49.4%	62.5%
Duration (years)	11.1	16.7	10.5
Schizophrenia	67.2%	45.6%	68.5%
Bipolar	14.3%	17%	13.9%
Depression	2.4%	7%	2%
GAF (mode)	55	60	55
CGI	4	4	4
Weight	79.3kg	85.0kg	78.6kg
BMI	26.4	29.4	26.1
Smokers	62%	66.7%	61.6%

### Prevalence of diabetes and characteristics

Using the old definition of diabetes incorporating IFG and OGTT, 62/798 (7.8%) had diabetes. The rate was 30/474 (6.3%) in men and 32/311 (10.3%) in women (Chi^2^ = 4.0, p = 0.04). Using a definition of diabetes, incorporating IFG, OGTT and HBA1c, 81/798 (10.2%) had diabetes; 8.2% in men and 13.2% in women (Chi^2^ = 5.3, p = 0.02). Compared to the non-diabetic group (n = 717, 62.5% male), there was a lower percentage of men in both the old (43.5%) and new definitions (49.4%). In addition, the patients in the old and new diabetes criteria both had higher BMI (29.7 and 29.4 respectively) than the non-diabetic patient group (26.1). There was a higher percentage of people with depression and bipolar disorder in the old and new diabetes criteria compared to the non-diabetic group but a higher percentage of people with schizophrenia in the non-diabetic group. Of 536 patients diagnosed with schizophrenia, diabetes was present in 6.3% (old definition) and 8.4% (new definition). The Cohen’s kappa agreement between the two definitions was 0.86 (95% CI = 0.78 to 0.93). The general European population rate of diabetes is approximately 3.3% (old definition) and 6.8% (new definition), but the patient group reported here has a significantly younger mean age.7 Correcting for this, the expected population rate would be 1.1% (old definition) and 1.4% (new definition) suggesting a relative risk of 7.1 and 7.3, respectively.

**Fig 1:**
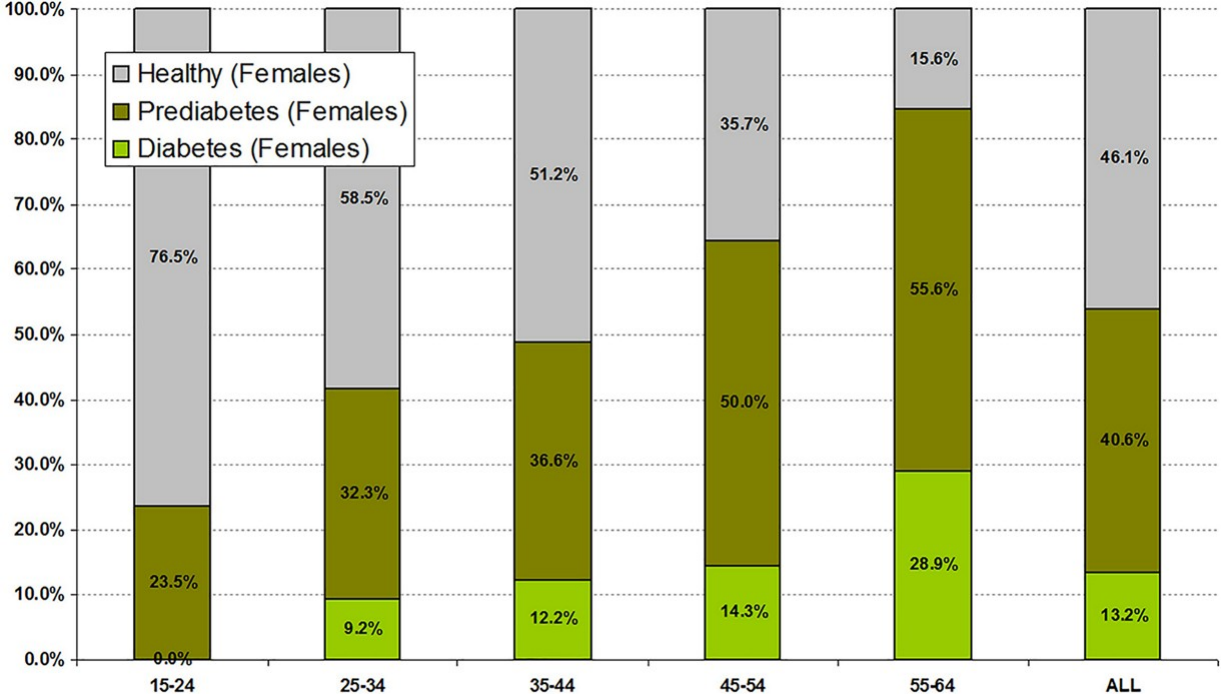
Prevalence rate of diabetes and prediabetes by age in women taking antipsychotics (n = 310).

**Fig 2:**
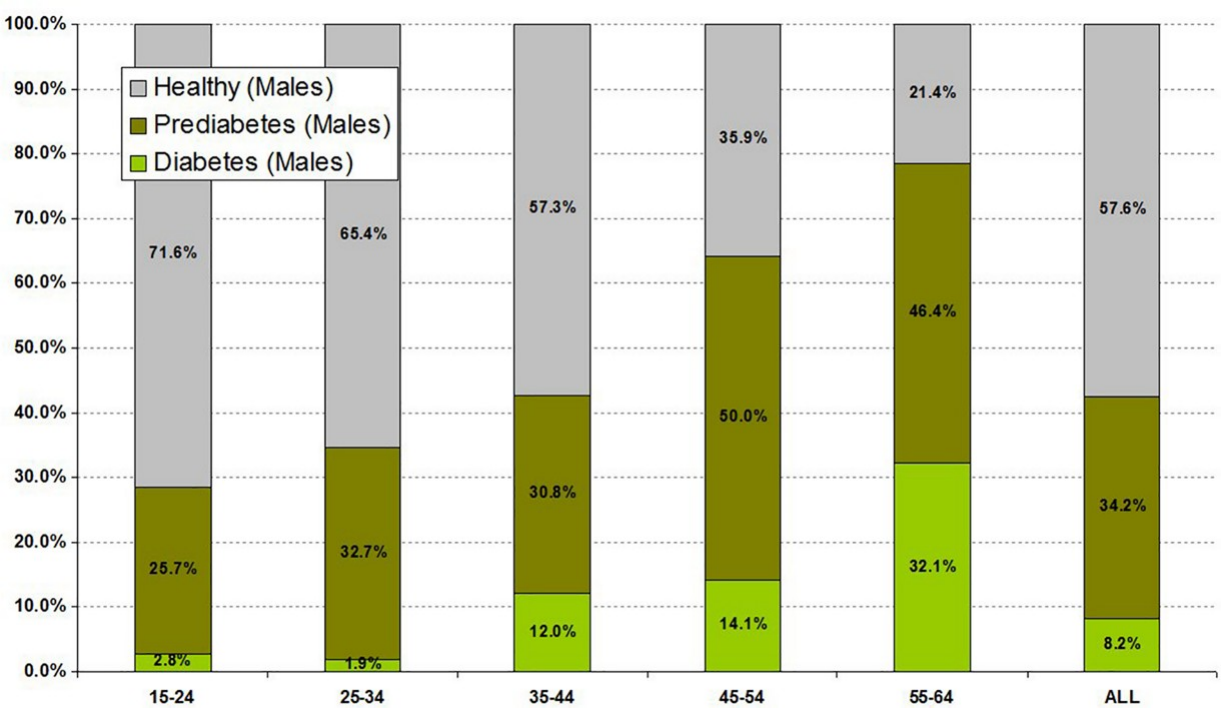
Prevalence rate of diabetes and prediabetes by age in men taking antipsychotics (n = 488).

Particularly high rates of diabetes were seen in some subgroups. The rate of old and newly defined diabetes in those taking antipsychotic drugs less than 3 months was 8.9% and 7.9%, 3–6 months 12.9% and 12.9%, 6–9 months 21.3% and 6.3%, and >1 year 10.4% and 13.1%, respectively. However, the highest rates related to age. In males and females aged 45–55 years old, diabetes was present in 14.1 and 14.3%, respectively, and in those aged 56–64, diabetes was present in 32.1% and 28.9% respectively (Figs 1 and 2).

### Glucose metabolism measurements

Mean cholesterol, trigyceride, HDL and LDL levels were 216 mg/dL, 229 mg/dL, 48 mg/dL, 124 mg/dL in the cohort of patients with diabetes using the old definition and 213 mg/dL, 211 mg/dL, 48 mg/dL and 124 mg/dL using the new definition. Using the old and new definitions of diabetes mean fasting glucose was 125.1 mg/dL vs. 117.4 mg/dL, mean fasting insulin was 20.9 mIU/Lvs. 20.1 mIU/L and mean HOMA-IR was 6.8 vs. 6.2, respectively. In short, the old definition of diabetes identified a more strongly at risk cohort according to their biochemical profile but the new definition identified a larger cohort at risk.

### Predictive accuracy of clinical variables in the diagnosis of diabetes

The performance of each individual clinical variable against biochemically defined diabetes is shown in Table 3. No clinical variable was particularly accurate. Judging by area under the curve, the most accurate variable was age (ROC = 0.742), followed by BMI (ROC = 0.653) and illness duration (ROC = 0.639). Judging by overall correct (defined as true positives + true negatives / all cases) the most accurate were BMI (76.6% correct), waist/hip ratio (74.2% correct) and height (68.7% correct). Rule-in and rule-out accuracy may be best considered separately. The optimal individual clinical variables to confirm (rule-in with minimal false positives) a diagnosis of diabetes were 1.) age and 2.) illness duration. However, no variable performed well, all were very poor at confirming a correct diagnosis. The optimal individual clinical variables to refute (rule-out with minimal false negatives) a diagnosis of diabetes were 1.) BMI 2.) waist/hip ratio and 3.) height. These three variables all performed well in this capacity and could be considered as initial screening questions to rule out people unlikely to have diabetes. For example, a BMI less than 30 would correctly identify non- diabetic patients with SMI with 92.5% accuracy (NPV) with 7.5% false negative rate. Of those non- diabetic patients with SMI 80% had a low BMI, but 20% had a high BMI despite being non-diabetic. However, a high BMI could not confirm a diagnosis. Only 45% of those with diabetes had a high BMI and of all patients with a high BMI, only 21.3% would actually be diabetic (PPV).

**Table 3:** Clinical factors in the diagnosis of diabetes in patients receiving antipsychotic medication.

	Sensitivity	Specificity	PPV	NPV	Clinical Utility (+)	Clinical Utility (-)	Overall Correct	AUC	Optimal Cut-Off ≥	LR(+)	LR -
Symptom	(95% CI)	(95% CI)	(95% CI)	(95% CI)	(95% CI)	(95% CI)				(95% CI)	(95% CI)
Individual Clinical Risk Factors											
Gender	54.0% (43.6%-64.5%)	62.3% (58.8%-65.9%)	14.6% (10.4%-18.7%)	92.0% (89.6%-94.3%)	Very poor (0.079, 0.076–0.081)	Fair (0.573, 0.572–0.574)	61.46	0.581 (0.526 to 0.637)	2	1.43 (1.16–178)	0.74 (0.58–0.93)
Age	80.5% (72.1%-88.8%)	58.7% (55.1%-62.2%)	18.8% (14.4%-23.2%)	96.2% (94.4%-98.0%)	Very poor (0.151, 0.148–0.154)	Fair (0.564, 0.563–0.565)	60.98	0.742 (0.689 to 0.794)	38.9	1.95 (1.70–2.23)	0.33 (0.22–0.51)
Mental health GAF	87.4% (80.4% - 94.3%)	20.2% (17.3%-23.1%)	11.5% (8.9%-14.1%)	93.1% (89.1%-97.0%)	Very poor (0.100, 0.099–0.102)	Very poor (0.188, 0.186–0.189)	27.32	0.504 (0.445 to 0.562)	45	1.09 (1.00–1.20)	0.63 (0.35–1.11)
Mental health CGI	93.1% (87.8%-98.4%)	13.0% (10.5%-15.4%)	11.3% (8.8%-13.7%)	94.1% (89.4%-98.7%)	Very poor (0.105, 0.103–0.106)	Very poor (0.122, 0.120–0.123)	21.46	0.508 (0.450 to 0.565)	5	1.07 (1.00–1.14)	0.53 (0.24–1.18)
SMI Duration Illness	72.4% (63.0%-81.8%)	56.9% (53.3%-60.5%)	16.6% (12.5%-20.7%)	94.6% (92.4%-96.7%)	Very poor (0.120, 0.118–0.123)	Fair (0.538, 0.537–0.539)	58.54	0.639 (0.571 to 0.706)	9.4	1.68 (1.44–1.96)	0.48 (0.34–0.69)
Height	49.4% (38.9%-59.9%)	70.9% (67.7%-74.2%)	16.8% (11.8%-21.8%)	92.2% (90.0%-94.4%)	Very poor (0.083, 0.080–0.086)	Good (0.654, 0.653–0.655)	68.66	0.621 (0.539 to 0.703)	1.68	1.70 (1.34–2.16)	0.71 (0.58–0.88)
Weight	71.3% (61.8%-80.8%)	42.0% (38.4%-45.6%)	12.7% (9.6%-15.9%)	92.5% (89.7%-95.3%)	Very poor (0.091, 0.089–0.093(	Poor (0.389, 0.387–0.390)	45.12	0.575 (0.511 to 0.639)	74.2	1.23 (1.06–1.42)	0.68 (0.49–0.96)
BMI	44.8% (34.4%-55.3%)	80.4% (77.5%-83.2%)	21.3% (14.6%-28.0%)	92.5% (90.4%-94.5%)	Very poor (0.096, 0.091–0.100)	Good (0.743, 0.742–0.744)	76.59	0.653 (0.590 to 0.716)	29.6	2.28 (1.73–3.00)	0.69 (0.57–0.83)
Waist/Hip Ratio	39.1% (28.8%-49.3%)	78.3% (75.3%-81.3%)	17.6% (11.7%-23.5%)	91.5% (89.4%-93.7%)	Very poor (0.069, 0.065–0.072)	Good (0.717, 0.716–0.718)	74.15	0.594 (0.528 to 0.661)	1	1.80 (1.34–2.42)	0.78 (0.65–0.92)
Combined Factors (Models)											
Leicester Practice Risk Score (LPRS) [cut off ≥ 14]	74.1% (64.5%-83.6%)	64.2% (60.6%-67.7%)	18.9% (14.1%-23.7%)	95.6% (93.8%-97.5%)	Very poor (0.140, 0.137–0.145)	Fair (0.614, 0.613–0.615)	65.16	0.756 (0.705 to 0.807)	14	2.07 (1.76–2.43)	0.40 (0.28–0.59)
TOPICS Model (TDRS) [cut off ≥ 8]	71.6% (61.8%-81.4%)	67.2% (63.8%-70.7%)	19.8% (14.7%-24.9%)	95.4% (93.6%-97.3%)	Very poor (0.142; 0.138–0.145)	Good (0.643, 0.641–0.643)	67.67	0.765 (0.712 to 0.817)	8	2.18 (1.84–2.60)	0.42 (0.30–0.60)

Footnote: AUC- Area under receiver operator characteristic curve; PPV–Positive predictive value; NPV—Negative predictive value; UI = Clinical utility index. The positive clinical utility index (UI+ = sensitivity x PPV) measures rule-in value and the negative clinical utility index (UI- = specificity x NPV) measures rule-out value. The following qualitative grades of diagnostic accuracy have been applied to the clinical utility index were > = 0.81: excellent, <0.81 good > = 0.64; <0.64 fair> = 0.49; <0.49 poor. > = 0.36; <0.36 very poor

### Predictive accuracy of clinical models in the diagnosis of diabetes

The performance of combined clinical variables in the Leicester Practice Risk Score (LPRS) and the Topics Diabetes Risk Score (TDRS) were evaluated against biochemically defined diabetes (Table 4). These clinical models were not particularly accurate but they were no less accurate than shown in their parent (non-SMI) studies (Table 1). Judging by area under the curve and by overall correct, the most accurate model was TDRS followed by LPRS. When rule-in and rule-out accuracy were considered separately, the optimal model to refute (rule-out with minimal false negatives) a diagnosis of diabetes was TDRS. When negative at a score of <8, TDRS had 95.4% negative predictive value, meaning only about 5% with a low score would be missed (false negatives). Although neither clinical model was satisfactory at confirming a diagnosis, either could be used as an initial screening model in order to rule out people unlikely to have diabetes. There was one limitation of this approach, TDRS and LPRS would only be negative (under threshold) in 67% and 64% of non-diabetic patients respectively. In this respect these combination models perform significantly worse than just considering BMI alone. A low BMI was almost as accurate as a low score on TDRS (92.5% vs 95.4%) but with the advantage that 80% of non-diabetic patients would have a relevant negative (under threshold) score compared with 67% for the TDRS. Thus, we conclude that these clinical prediction models have little if any advantage over the individual clinical variables used alone. For completeness, we finally examined the accuracy of the clinical models to identify either pre-diabetes or diabetes. Accuracy was inferior when identifying both pre-diabetes and diabetes compared with diabetes alone. For either pre-diabetes or diabetes the AUC for TDRS was 0.661 (CI = 0.624 to 0.699) compared with 0.765 (CI = 0.712 to 0.817) for diabetes alone. For either pre-diabetes or diabetes the AUC for LPRS was 0.647 (CI = 0.609 to 0.685) compared with 0.756 (CI = 0.705 to 0.807) for diabetes alone. This confirms that these models were not particularly suitable for the identification of pre-diabetes or diabetes in SMI.

**Table 4:** Optimal biochemical measure vs old definition of diabetes.

	Sensitivity	Specificity	PPV	NPV	Clinical Utility (+)	Clinical Utility (-)	Overall Correct	AUC	Optimal Cut-Off ≥	LR(+)	LR -
symptom	**(95% CI)**	**(95% CI)**	**(95% CI)**	**(95% CI)**	**(95% CI)**	**(95% CI)**				**(95% CI)**	**(95% CI)**
HBA1C	80.6% (70.8–90.5)	71.3% (68.1–74.6)	19.2% (13.9–24.5)	97.8% (96.5–99.0)	Very Poor 0.154 (0.150–0.159)	Good 0.697 (0.697–0.698)	72.06	0.832 (0.778–0.887)	5.8	2.81	0.27 (0.16–0.45)
Fasting Glucose	91.9% (85.5–98.7)	82.5% (79.7–85.2)	30.6% (22.7–38.6)	99.2% (98.5–99.9)	Very Poor 0.282 (0.267–0.288)	Excellent 0.818 (0.818–0.818)	83.21	0.935 (0.894–0.975)	98 mg/dl	5.25	0.10 (0.04–0.23)
OGTT 30min	85.5% (76.7–94.3)	76.7% (73.6–79.8)	24.0% (17.5–30.4)	98.4% (97.4–99.4)	Very Poor 0.205 (0.200–0.210)	Good 0.755 (0.754–0.755)	77.39	0.867 (0.817–0.918)	179 mg/dl	3.67	0.19 (0.10–0.35)
OGTT 60min	91.9% (85.2–98.7)	83.5% (80.8–86.2)	32.4% (24.0–40.8)	99.2% (98.5–99.9)	Very Poor 0.298 (0.291–0.304)	Excellent 0.828 (0.828–0.829)	84.16	0.936 (0.900–0.971)	181 mg/dl	1.10	0.10 (0.04–0.22)
OGTT 120min	88.7% (80.8–96.6)	91.1% (89.0–93.2)	46.2% (34.1–58.4)	98.9% (98.2–99.7)	Poor 0.410 (0.402–0.418)	Excellent 0.902 (0.901–0.902)	90.93	0.947329 (0.908–0.986)	145 mg/dl	0.97	0.12 (0.06–0.25)
Fasting Insulin	56.5% (44.1–68.8)	81.9% (79.1–84.7)	20.8% (13.9–27.7)	95.7% (94.1–97.3)	Very Poor 0.118 (0.112–0.123)	Good 0.784 (0.784–0.785)	79.95	0.722 (0.645–0.799)	14.7	3.12	0.53 (0.40–0.71)
HOMA-IR	66.1% (54.3–77.9)	86.7% (84.2–89.1)	29.5% 20.5–38.5)	96.8% (95.5–98.2)	Very Poor 0.195 (0.188–0.202)	Excellent 0.839 (0.839–0.840)	85.09	0.800 (0.733–0.866)	3.38	4.97	0.39 (0.28–0.55)
Apriori Thresholds											
HBA1C ≥6.5	27.4% (16.3–38.5)	97.4% (96.3–98.6)	47.2 (24.2–69.6)	94.1% (92.4–97.2)	Very Poor 0.129 (0.117–0.142)	Excellent 0.917 (0.916–0.917)	91.98	0.624 (0.568–0.680)	≥6.5	N/A	0.75 (0.64–0.87)
Fasting Glucose >125mg/dl	48.4% (35.9–60.8)	100.0% (100–100)	100.0% (100–100)	95.8% (94.4–97.2)	Poor 0.484 (0.469–0.499)	Excellent 0.958 (0.958–0.958)	95.99	0.742 (0.679–0.804)	>125mg/dl	N/A	0.52 (0.41–0.66)
OGTT 120min ≥199mg/dl	74.2% (63.3–85.1)	100.0% (100–100)	100.0% (100–100)	97.9% (96.8–98.9)	Good 0.742 (0.734–0.750)	Excellent 0.979 (0.979–0.979)	97.99	0.871 (0.816–0.925)	≥199mg/dl	N/A	0.26 (0.17–0.39)
Algorithm Approaches											
Van Winkel (FG then OGTT)	59.7% (47.5–71.9)	100.0% (100–100)	100.0% (100–100)	96.7% (95.4–98.0)	Fair 0.597 (0.584–0.609)	Excellent 0.967 (0.967–0.967)	96.87	0.798 (0.736–0.860)	As per algorithm	N/A	0.40 (0.30–0.55)
Mitchell(a) (HBA1c > FG AND OGTT)	71.0% (59.7–82.3)	100.0% (100–100)	100.0% (100–100)	97.6% (96.5–98.7)	Good 0.710 (0.701–0.719)	Excellent 0.976 (0.976–0.976)	97.74	0.855 (0.798–0.911)	As per algorithm	N/A	0.29 (0.20–0.43)
Mitchell(b) (HBA1c > OGTT)	54.8% (42.5–67.2)	100.0% (100–100)	100.0% (100–100)	96.3% (95.0–97.7)	Fair 0.548 (0.535–0.562)	Excellent 0.963 (0.963–0.963)	96.49	0.774 (0.712–0.837)	As per algorithm	N/A	0.45 (0.34–0.59)
Mitchell(c) (HBA1c> FG)	33.9% (22.1–45.7)	100.0% (100–100)	100.0% (100–100)	94.7% (93.2–96.3)	Very Poor 0.339 (0.321–0.357)	Excellent 0.947 (0.947–0.947)	94.86	0.669 (0.610–0.729)	As per algorithm	N/A	0.66 (0.55–0.79)
Mitchell(d)(HBA1c > FG or OGTT)	45.2% (32.8–57.5)	100.0% (100–100)	100.0% (100–100)	95.6% (94.1–97.0)	Poor 0.453 (0.436–0.468	Excellent 0.956 (0.956–0.956)	95.74	0.589 (0.541–0.637)	As per algorithm	N/A	0.55 (0.44–0.69)
Mitchell(e)(HBA1c5.8 >ALL)	71.0% (59.7–82.3)	100.0% (100–100)	100.0% (100–100)	97.6% (96.4–98.7)	Good 0.710 (0.701–0.719)	Excellent 0.976 (0.975–0.976)	97.69	0.854 (0.798–0.912)	As per algorithm	N/A	0.29 (0.20–0.43)
Mitchell(f)(HBA1c5.7 >ALL)	80.6% (70.8–90.5)	100.0% (100–100)	100.0% (100–100)	98.4% (97.5–99.3)	Good 0.806 (0.800–0.813)	Excellent 0.984 (0.984–0.984)	98.5	0.903 (0.854 to 0.953)	As per algorithm	N/A	0.19 (0.12–0.32)

Footnote: AUC- Area under receiver operator characteristic curve; PPV–Positive predictive value; NPV—Negative predictive value; UI = Clinical utility index. The positive clinical utility index (UI+ = sensitivity x PPV) measures rule-in value and the negative clinical utility index (UI- = specificity x NPV) measures rule-out value. The following qualitative grades of diagnostic accuracy have been applied to the clinical utility index were > = 0.81: excellent, > = 0.64: good and > = 0.49: fair <0.49 = poor.OGTT–oral glucose tolerance test. Algorithm approaches are as follows:Van Winkel (FG >100mg/dl then OGTT?199mg/dl); Mitchell(a) (HBA1c >5.8 then IFG AND OGTT); Mitchell(b) (HBA1c >5.8 then OGTT); Mitchell(c) (HBA1c >5.8 then IFG); Mitchell(d) (HBA1c >5.8 then IFG or OGTT); Mitchell(e) (HBA1c >5.8 then IFG and OGTT and HBA1c)

### Predictive accuracy of HBA1c and related variables for Pre2010 diabetes definition

#### Single metabolic tests

**Fig 3:**
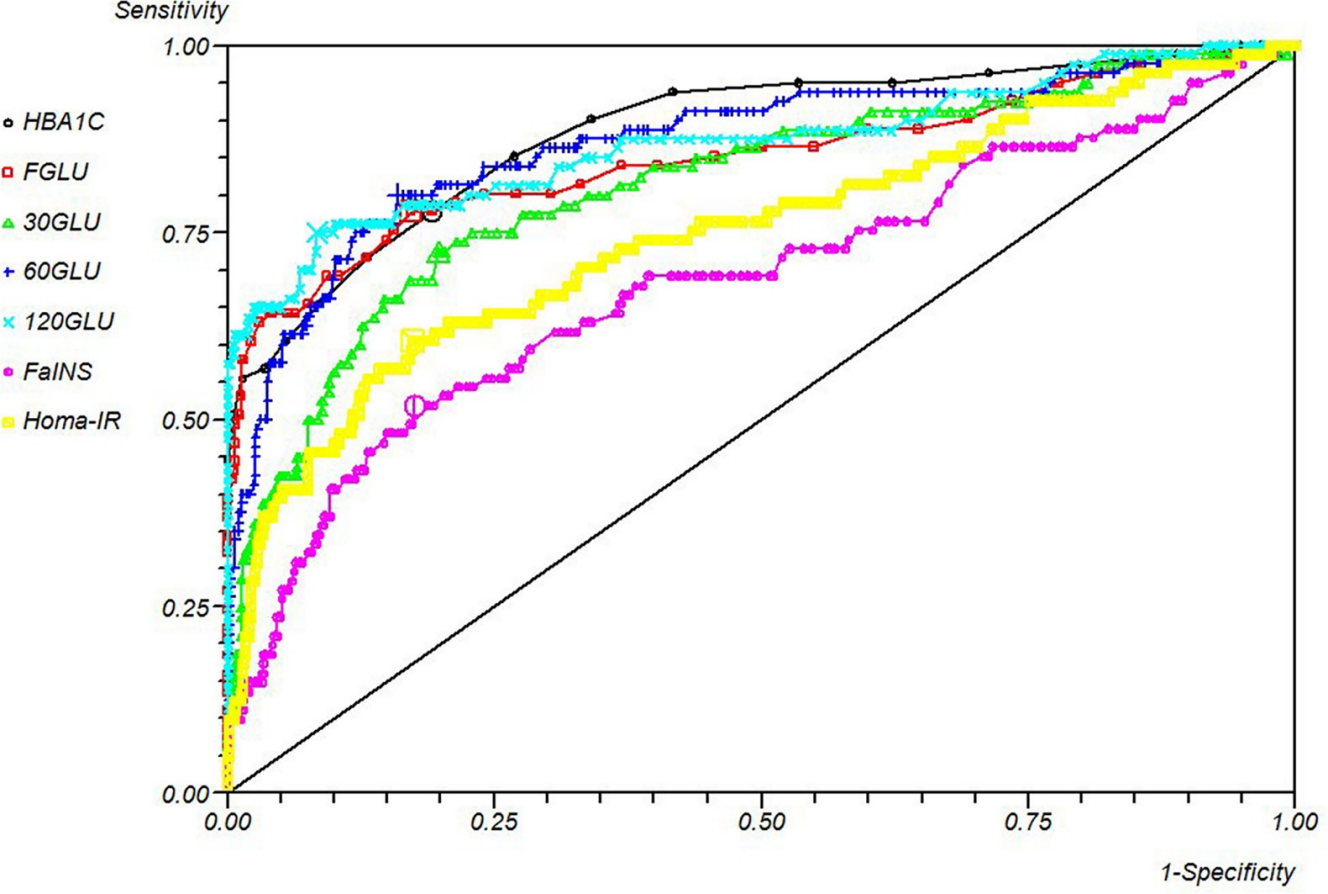
ROC curve of metabolic measures vs old definition of diabetes.

The performance of metabolic biochemical markers against the pre-2010 (conventional) definition of diabetes is shown in Table 2 and Fig 3. After choosing the optimal cut, no test was satisfactory for confirming diabetes. The best single test that could be used to confirm a diagnosis of diabetes with minimal false positives was the two-hour OGTT which had a positive predictive value (PPV) of 46%. All tests performed better in a rule-out capacity, which excludes those without a diagnosis of diabetes with minimal false negatives. All tests had a negative predictive value (NPV) above 95%, but the one-hour response to OGTT and fasting glucose (at a cut-point of 98mg/dl) both had NPV’s above 99%. However, both these tests suffered from a relative lack of specificity. When seeking the optimal rule-out test, the discrimination and occurrence of a negative test in those without the condition should be considered equally. With this in mind, the optimal rule-out test was the two-hour OGTT result, followed by HOMA-IR, fasting glucose level and one-hour OGTT. HBA1c offered a very poor confirmation of diabetes with only 19% PPV, but it could be used as part of a screening algorithm (see below), as it possessed 97.8% NPV.

#### Single metabolic tests (Apriori thresholds)

Using conventional cut-offs proposed for the diagnosis of diabetes in the general population revealed some unexpected findings. Conventional cut-offs in HBA1c, fasting glucose or OGTT all produced excellent rule-out statistics with few false negatives, suitable for use as an initial screening step. However, only two-hour OGTT (at >199mg/dl) was satisfactory for case-finding of diabetes. Even in this case, only 3 in 4 of subjects with diabetes had an OGTT >199mg/dl; 1 in 4 would be missed (sensitivity was 74%). HBA1c at the conventional cut-point of ≥6.5 proved wholly unsatisfactory as a method of confirming (older) diabetes. Only about 1 in 4 with diabetes would score positive on HBA1c at a cut-point of 6.5% (48 mmol/mol), 3 in 4 being missed.

#### Algorithm approaches

Regarding aim 2 of the study, we investigated seven algorithm approaches to the diagnosis of diabetes. The algorithm proposed by van Winkel [20], namely a fasting glucose >100mg/dl followed by a conventional OGTT for patients positive in step 1, gave 100% specificity and PPV and very good NPV (96.7%) suggesting a potentially useful approach. Its main limitation was the sensitivity of only 59.7% meaning 4 in 10 people with diabetes would potentially be missed. A more convenient screening algorithm “Mitchell (b)” consisting of (HBA1c >5.8% then OGTT) offered almost identical accuracy as the van Winkel proposal, but without requiring an initially fasting sample. “Mitchell (b)” would necessitate OGTT in only 25% of patients. The van Winkel algorithm would require a fasting sample on everyone, but OGTT in only 20% of patients [20]. The only approaches that offered good or better clinical utility were an initial HBA1c at a lower threshold (≥5.9 or ≥5.7) followed by conventional diabetes testing with the addition of IFG and OGTT. For maximum accuracy, this protocol requires patients initially screening positive to have IFG and OGTT as well as reinterpretation of HBA1c ≥6.5. At a HBA1c cut-off of ≥5.9, this protocol achieves 97.7% overall accuracy, but only requires OGTT and fasting glucose in 25% of the sample, and at a cut-off of ≥5.7 this protocol achieves 98.5% overall accuracy and only requires OGTT and fasting glucose in 32.7% of the sample as opposed to 100% testing of all three measures to achieve 100% accuracy. The latter strategy achieves 80.6% sensitivity and 100% specificity.

### Predictive accuracy of biochemical variables for detecting diabetes (2010 Diabetes definition)

#### Single metabolic tests

**Fig 4:**
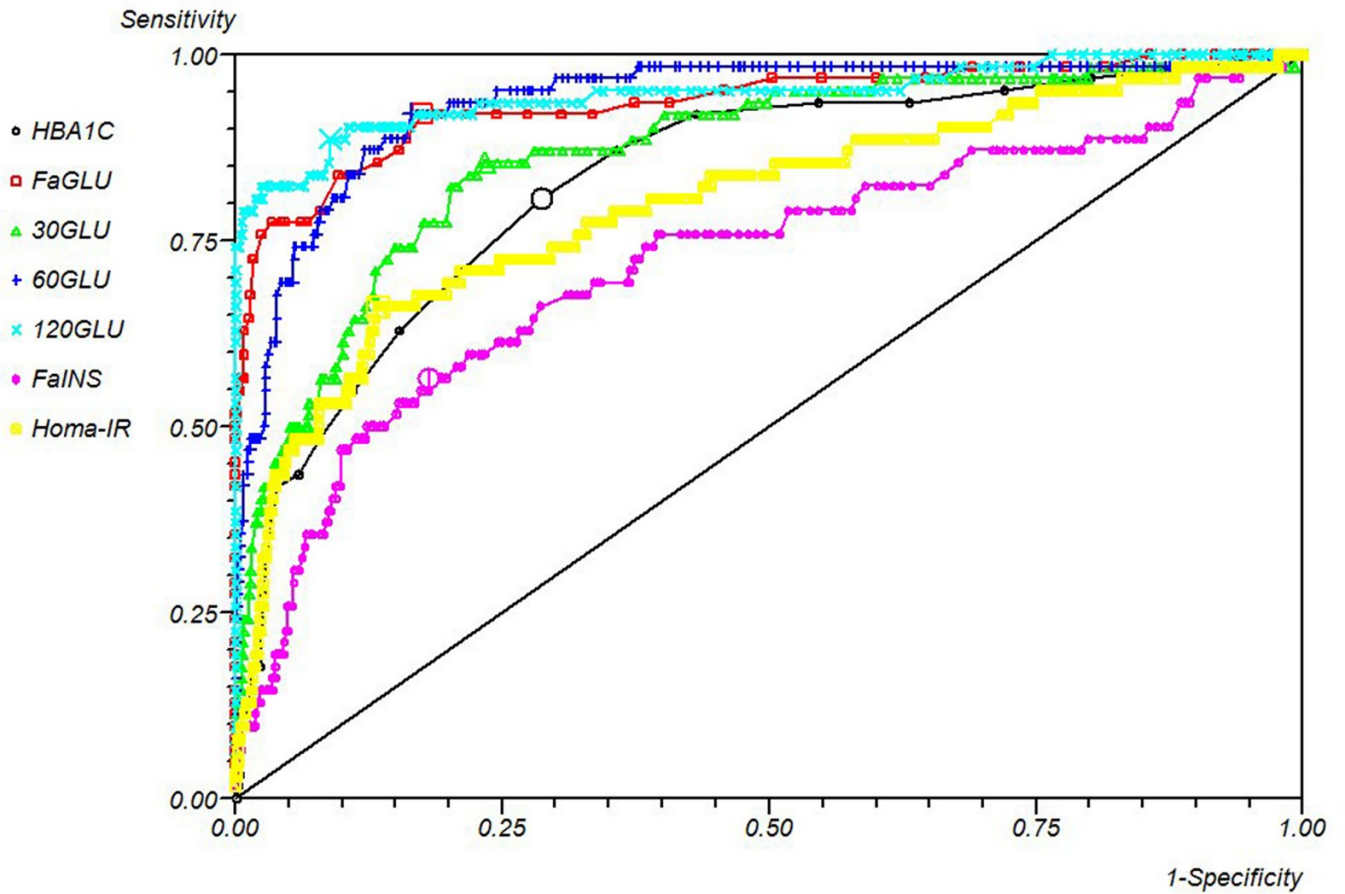
ROC curve of metabolic measures vs new definition of diabetes.

The performance of metabolic biochemical markers against a close adaption of the 2010 definition of diabetes is shown in Table 5 and Fig 4. All tests were disappointing in their ability to diagnose diabetes when used alone. OGTT (at ≥145 mg/dl) had 50% PPV and HBA1c only 31.5%. As above, all tests performed better in a rule-out capacity that excludes those without a diagnosis of diabetes with minimal false negatives. All tests had NPVs above 90%, but the highest values were those related to the OGTT, fasting glucose with HBA1c. When combined with their occurrence in patients without diabetes, the optimal screening test was an adapted OGTT. Judging the overall performance by fraction/overall correct: OGTT was the optimal single test.

**Table 5:** Optimal metabolic measure vs new definition of diabetes.

Symptom	Sensitivity (95% CI)	Specificity(95% CI)	PPV(95% CI)	NPV(95% CI)	Clinical Utility (+)(95% CI)	Clinical Utility (-)(95% CI)	Overall Correct	AUC(95% CI)	Optimal / Preselected Cut-Off ≥	LR+(95% CI)	LR-(95% CI)
Single Metabolic Tests											
HBA1C	77.8% (68.7–86.8)	80.9% (78.0–83.8)	31.5% (23.7–39.3)	97.0% (95.6–98.4)	Very Poor 0.245 (0.240–0.250)	Good 0.785 (0.784–0.785)	80.58	0.885 (0.841–0.930)	5.8	4.07 (3.37–4.92)	0.27 (0.18–0.41)
Fasting Glucose	77.8% (68.7–86.8)	82.8% (80.1–85.6)	33.9% (25.5–42.4)	97.1% (95.7–98.4)	Very Poor 0.263 (0.258–0.269)	Good 0.804 (0.804–0.805)	82.33	0.849 (0.791–0.907)	98 mg/dl	4.53 (3.72–5.53)	0.27 (0.18–0.40)
OGTT 30min	72.5% (62.7 = 82.3)	80.2% (77.3–83.2)	29.4% (21.9–37.0)	96.2% (94.7–97.8)	Very Poor 0.213 (0.208–0.219)	Good 0.772 (0.772–0.773)	79.44	0.814 (0.759–0.870)	179 mg/dl	3.67 (3.00–4.48)	0.34 (0.24–0.49)
OGTT 60min	80.0% (71.2–88.8)	84.1% (81.4–86.8)	36.4% (27.5–45.2)	97.4% (96.1–98.6)	Very Poor 0.291 (0.285–0.297)	Excellent 0.819 (0.818–0.819)	83.65	0.869 (0.819–0.919)	181 mg/dl	5.02 (4.10–6.15)	0.24 (0.15–0.37)
OGTT 120min	75.0% (65.5–84.5)	91.6% (89.6–93.7)	50.4% (37.7–63.1)	97.0% (95.7–98.3)	Poor 0.378 (0.371–0.386)	Excellent 0.888 (0.888–0.889)	89.91	0.870 (0.816–0.923)	145 mg/dl	8.94 (6.79–1.77)	0.27 (0.19–0.40)
Fasting Insulin	51.9% (41.0–62.7)	82.4% (79.6–85.2)	25.0% (17.5–32.5)	93.8% (91.9–95.7)	Very Poor 0.130 (0.125–0.135)	Good 0.773 (0.773–0.774)	79.32	0.685 (0.614–0.755)	14.7	2.95 (2.27–3.84)	0.58 (0.46–0.73)
HOMA-IR	60.5% (49.8–71.1)	82.7% (79.9–85.5)	28.3% (20.4–36.2)	94.9% (93.2–96.6)	Very Poor 0.171 (0.166–0.177)	Good 0.785 (0.784–0.785)	80.45	0.748 (0.683–0.813)	3.38	3.50 (2.76–4.44)	0.48 (0.36–0.63)
Apriori Thresholds											
HBA1C ≥6.5	44.4% (33.6–55.3)	100.0% (100–100)	100.0% (100–100)	94.1% (92.4–95.8)	Poor 0.444 (0.432–0.457)	Excellent 0.941 (0.941–0.941)	94.36	0.722 (0.667–0.776)	≥6.5	N/A	0.56 (0.46–0.68)
Fasting Glucose >125mg/dl	37.0% (26.5–47.6)	100.0% (100–100)	100.0% (100–100)	93.4% (91.6–95.1)	Poor 0.370 (0.357–0.384)	Excellent 0.934 (0.933–0.934)	93.61	0.685 (0.632–0.738)	>125mg/dl	N/A	0.63 (0.53–0.74)
OGTT 120min ≥199mg/dl	56.8% (46.0–67.6)	100.0% (100–100)	100.0% (100–100)	95.3% (93.8–96.8)	Fair 0.568 (0.558–0.578)	Excellent 0.953 (0.953–0.954)	95.61	0.784 (0.730–0.838)	≥199mg/dl	N/A	0.43 (0.34–0.55)
Algorithm Approaches											
Van Winkel (FG then OGTT)	45.7% (34.8–56.5)	100.0% (100–100)	100.0% (100–100)	94.2% (92.6–95.9)	Poor 0.457 (0.445–0.469)	Excellent 0.942 (0.942–0.942)	94.49	0.728 (0.717–0.826)	As per algorithm	N/A	0.54 (0.44–0.66)
Mitchell(a) (HBA1c > FG AND OGTT)	54.3% (43.5–65.2)	100.0% (100–100)	100.0% (100–100)	95.1% (93.6–96.6)	Fair 0.543 (0.533–0.554)	Excellent 0.951 (0.951–0.951)	95.36	0.772 (0.717–0.826)	As per algorithm	N/A	0.46 (0.36–0.58)
Mitchell(b) (HBA1c > OGTT)	42.0% (31.2–52.7)	100.0% (100–100)	100.0% (100–100)	93.8% (92.1–95.6)	Poor 0.420 (0.407–0.433)	Excellent 0.938 (0.938–0.939)	94.11	0.710 (0.656–0.764)	As per algorithm	N/A	0.58 (0.48–0.70)
Mitchell(c) (HBA1c> FG)	25.9% (16.4–35.5)	100.0% (100–100)	100.0% (100–100)	92.3% (90.4–94.2)	Very Poor 0.259 (0.245–0.274)	Excellent 0.923 (0.923–0.923)	92.48	0.629 (0.582–0.678)	As per algorithm	N/A	0.74 (0.65–1.18)
Mitchell(d)(HBA1c > FG or OGTT)	34.6% (24.2–44.9)	100.0% (100–100)	100.0% (100–100)	93.1% (91.3–94.9)	Very Poor 0.346 (0.332–0.360)	Excellent 0.931 (0.931–0.931)	93.36	0.568 (0.530–0.605)	As per algorithm	N/A	0.65 (0.56–0.77)
Mitchell(e)(HBA1c5.8>ALL)	77.8% (68.7–86.8)	100.0% (100–100)	100.0% (100–100)	97.6% (96.4–98.7)	Good 0.778 (0.772–0.783)	Excellent 0.976 (0.975–0.976)	97.74	0.889 (0.843–0.934)	As per algorithm	N/A	0.22 (0.15–0.33)
Mitchell(f)(HBA1c5.7 >ALL)	85.2% (77.4–92.9)	100.0% (100–100)	100.0% (100–100)	97.6% (96.4–98.7)	Excellent 0.852 (0.848–0.855)	Excellent 0.984 (0.983–0.984)	98.50	0.925 (0.887 to 0.965)	As per algorithm	N/A	0.15 (0.09–0.25)

Footnote: AUC- Area under receiver operator characteristic curve; PPV–Positive predictive value; NPV—Negative predictive value; UI = Clinical utility index. The positive clinical utility index (UI+ = sensitivity x PPV) measures rule-in value and the negative clinical utility index (UI- = specificity x NPV) measures rule-out value. The following qualitative grades of diagnostic accuracy have been applied to the clinical utility index were > = 0.81: excellent, > = 0.64: good and > = 0.49: fair <0.49 = poor.OGTT–oral glucose tolerance test. Algorithm approaches are as follows: Van Winkel (FG >100mg/dl then OGTT?199mg/dl); Mitchell(a) (HBA1c >5.8 then IFG AND OGTT); Mitchell(b) (HBA1c >5.8 then OGTT); Mitchell(c) (HBA1c >5.8 then IFG); Mitchell(d) (HBA1c >5.8 then IFG or OGTT); Mitchell(e) (HBA1c ?5.9 then IFG and OGTT and HBA1c) and Mitchell(f) (HBA1c >5.7 then IFG and OGTT and HBA1c)

#### Single metabolic tests (a priori thresholds)

Using conventional cut-offs in HBA1c by definition produced a correct confirmation of diabetes. In addition, all produced excellent rule-out statistics with few false negatives, suitable for use as an initial screening step. However, only 2hr OGTT can be seriously considered for case-finding newly defined diabetes in those taking antipsychotic medication. Here, although an OGTT >199 would define diabetes (100% PPV), such a result would only occur in 57% of people with diabetes. HBA1c was unsatisfactory as a method of confirming newly redefined diabetes, as only 4 out of 10 patients with diabetes would score positive on HBA1c at a cut-point of 6.5% (48 mmol/mol).

#### Algorithm approaches

We investigated the same seven algorithm approaches to the diagnosis of newly defined diabetes. The most accurate approach was an initial HBA1c followed by conventional testing. The main question is what cut-off on HBA1c is optimal. At a cut-off of ≥5.9 this protocol achieves 97.7% overall accuracy, and only requires OGTT and fasting glucose in 25% of the sample, and at a cut-off of ≥5.7, this protocol achieves 98.5% overall accuracy, and only requires OGTT and fasting glucose in 32.7% of the sample as opposed to 100% testing of all three measures to achieve 100% accuracy. Its only limitation was a slight loss in sensitivity to 85.2%. As the cut-off of ≥5.7 is also now recommended for prediabetes, this is the one we would recommend in SMI.

## Discussion

Diabetes is increasingly recognised as important in patients with SMI. Observational studies have reported a clear association between patients with established severe mental illness and diabetes [8, 44, 45]. The risk appears particularly severe in those maintained on antipsychotic drugs, notably most atypical antipsychotics although risk is elevated in those older conventional antipsychotics [46, 47]. A series of meta-analyses have documented that the rate of diabetes is high in those with chronic schizophrenia (12.8%; N = 9; n = 2142; 39.3±9.4 yrs), bipolar disorder (9.0%; N = 4; n = 1118; 42.1±8.5 yrs) and depression (7.6%; N = 6; n = 2827; 47.1±7.6 yrs) [48, 49, 50]. Pre-diabetes defined by impaired fasting glucose > 100mg/dl was also high in schizophrenia (24.2%) bipolar disorder (17.3%) and depression (17.6%). In short, all those with SMI appear to have a high risk of pre-diabetes and diabetes. This is particularly the case for those maintained on long-term atypical antipsychotic medication [44]. There is therefore a great need to identify and treat glucose dysregulation in patients on typical and non-atypical antipsychotics. Unfortunately it is also clear that the implementation of screening for diabetes and metabolic components is inconsistent [51, 52]. Even after the launch of many national guidelines on physical healthcare monitoring, blood glucose is only tested in about half of patients under psychiatric care [43]. Rates of metabolic surveillance appear to be significantly lower for patients with SMI than for patients with known diabetes [53, 54]. Indeed biochemical tests are often inadequately collected in patients with SMI. This may be particularly the case for patients in prison, in long-stay facilities, those seen in primary care and also those seen at home and in general medical hospital settings [55]. Yet, non-invasive clinical tests are often more frequently offered. A recent meta-analysis showed that 75% of patients with SMI on antipsychotic medication received assessment of body weight. Whilst 75% is still less than ideal, many clinicians consider measurement of clinical variables as practical and measurable in busy settings but would consider measurement of biochemical variables impractical.

This is the first study to examine whether clinical variables can be used to identify patients at high risk of diabetes in mental health settings. None of the clinical variables: gender, age, mental health global assessment of function (GAF), mental health clinicians’ global impression (CGI), duration of severe mental illness, height, weight, BMI, waist/hip ratio were completely satisfactory and none could be used instead of conventional biochemical testing. Where biochemical testing is considered impractical or inconvenient (or perhaps when facilities are not available) BMI or waist/hip ratio could be used as an approximate initial screen in order to rule-out those at low risk. A BMI less than 30 would correctly identify non-diabetic patients with SMI with about 92.5% accuracy (NPV), that is, with a 7.5% false negative rate. BMI could not be used to confirm the presence of diabetes because of its low PPV (21.3%). Given the high rates of not just diabetes, but also pre-diabetes in SMI, there may be some merit in screening for pre-diabetes and diabetes combined. If the BMI was used to identify not just diabetes but pre-diabetes and diabetes, then the PPV would increase from 21% to 65.2% but NPV would fall to from 92.5% to 58.5%. Thus performance of the optimal clinical variable (BMI) would remain unsatisfactory even if pre-diabetes was the target. This means that basing judgments about diabetes (or pre-diabetes) upon single clinical factors cannot be recommended.

The next question we addressed is whether a combination of clinical variables can be used to identify patients at high risk of diabetes in mental health settings. Previous studies appear to suggest that combination models may improve upon the accuracy offered by individual clinical variables alone. Based on the published literature regarding the performance of clinical models in the general population (Table 1) we chose to examine the Leicester Practice Risk Score (LPRS) and the Topics Diabetes Risk Score (TDRS) in patients with SMI. In large scale population studies the LPRS achieved an area under the curve 0.72 and a Youden score of 0.239.30 The TDRS achieved an area under the curve of 0.77 and a Youden score of 0.408 [31]. In this study, we found very similar results. Here the LPRS achieved an area under the curve 0.756 and a Youden score of 0.383. The TDRS achieved an area under the curve of 0.765 and Youden score of 0.388. Thus the performance of these models was almost identical to their own validation cohorts and indeed the recommended cut points were the same on ROC curve testing. Yet in practical terms, neither could be used to reliably confirm diabetes. A negative TDRS (a score of <8) was potentially useful in that the TDRS had 95.4% negative predictive value (yielding 5% false negatives) but the TDRS would only be negative (under threshold) in 67% of non-diabetic SMI patients. Further when looking for at risk patients (with either pre-diabetes or diabetes) their performance was further reduced. Overall then the clinical prediction models appear to have little if any advantage over the individual clinical variables used alone.

The prompt detection of diabetes is a priority in patients with SMI but we find little to support the routine use of clinical variables in order to accurately identify those with diabetes (or indeed at risk with prediabetes). It is possible that clinical risk factors of importance were not measured in this study and the future risk profiling may prove beneficial. For example several models incorporated diet and fitness, not measured in this study. In this study age, BMI, waist/hip ratio and illness duration were associated with diabetes and could be incorporated informally by clinicians concerned about risk of diabetes in order to focus advice or resources. However, no clinical variables were a satisfactory proxy for a diagnosis of diabetes and as such we recommend conventional biochemical testing for patients with SMI where diabetes or prediabetes is a potential concern.

Regarding biochemical predictors, we found that a single application of HBA1c and other markers of glucose regulation should be used with caution in patients with SMI taking antipsychotics. Repeat testing (after several weeks) using the HBA1c would likely improve accuracy but this has not been formally studied even in the general population [56]. Since this study was conducted before the introduction of 2010 guidelines we could not precisely replicate the 2010 recommendations. Nevertheless results of this large prospective observation study demonstrate that in settings using the older definition of diabetes, the two-hour OGTT is the optimal single test at a cut point of 199 mg/dL, but this is not perfect, as reliance on this one test would miss 25.8% of diabetic cases. At an a-priori cut of 199 mg/dL (7.0mol/L), there was excellent rule-out ability, but a reduced performance for case-finding diabetes, simply because one in four people with diabetes have a normal OGTT, but abnormal fasting glucose. However, reliance on IFG (at a cut point of >125 mg/dL) would miss 51.6% of people with diabetes and reliance on HBA1c (at a cut point of ≥6.5% (48 mmol/mol)) would miss 72.6% of people with diabetes. This means that the gold standard of a fasting glucose and an OGTT cannot be replaced by a single test if optimal accuracy is required. Regarding metabolic tests for the newly proposed definition of diabetes, the same limitations apply. Conventional OGTT, IFG and HBA1c miss 43.2%, 67% and 55.6% people with diabetes respectively when used alone. HBA1c is sometimes proposed as a single one-off test of diabetes in some centres. We have shown this is not recommended at the conventional cut-point of ≥6.5% (48 mmol/mol) in this population due to its poor sensitivity. Only about 4 in 10 patients with diabetes score at 6.5% (48 mmol/mol) or above. In clinical practice, this would result in the majority of people with diabetes patients being missed if this were the only test used. HbA1c is generally less sensitive than IFG and OGTT in diagnosing diabetes in those with mild disease [8].

**Fig 5:**
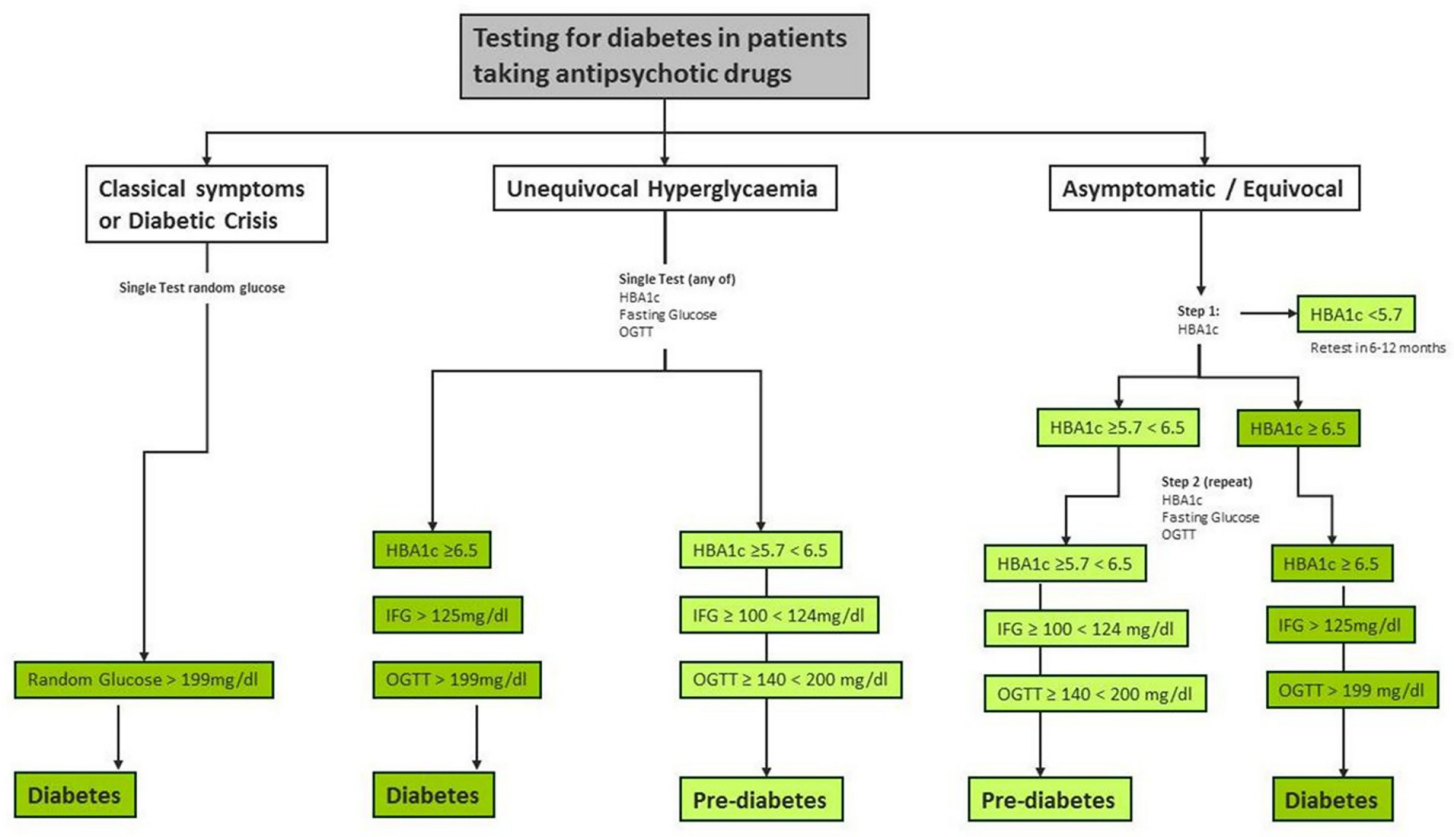
Optimal protocol for testing diabetes in SMI.

We found that HBA1c can be used as an initial screening step in a diagnostic algorithm (Fig 5). An algorithm consisting of HBA1c ≥5.7% followed by both FG and OGTT was the optimal test that did not require all patients to have HBA1c, OGTT and FG. It is important to note that modification of the cut-point to 5.7% improves sensitivity from 44.4% to up to 85%. A higher cut-point of ≥5.9% could be chosen, but at the penalty of loss in sensitivity. At ≥5.7% only one in three people would need to have fasting and challenge test. Therefore, in psychiatric settings, for patients with SMI taking antipsychotics we recommend a modification of the cut-point to ≥5.7% as defined by the newly defined World Health Organisation/American Diabetes Association standard [57] when looking for diabetes. We believe these results are generalisable to most organizations treating patients with antipsychotic medication. An HBA1c cut-point of ≥5.7% is identical to the one found using ROC analyses of the US NHANES data (≥5.7%) as the best combination of sensitivity (39%) and specificity (91%) to identify pre-diabetes [58]. HBA1c ≥5.7% was subsequently adopted internationally as the threshold to diagnose prediabetes. Several previous studies measured HBA1c in patients with SMI taking antipsychotics. Krein et al (2006) found mean HbA1c to be lower among those with versus those without SMI, although testing was not systematic in this study [59]. Brown et al (2011) found that diabetic patients with SMI had lower HbA1c levels than those without SMI [60]. In this context, second-generation antipsychotics appear to adversely influence HBA1c levels [61].

### Recommendations for application of a diagnostic test for diabetes

Taking results together we suggest the following approach. All patients with SMI taking antipsychotic medications at initiation of the drug should be tested using HBA1c ≥5.7%. If negative, the test should be repeated again in 6 months. If positive, proceed to step 2, immediately (but certainly within 2 weeks) obtain and use HBA1c and fasting glucose and OGTT at conventional cuts-offs. If testing is done immediately, HBA1c does not need to be repeated, as those scoring ≥6.5% (48 mmol/mol) are already apparent. OGTT and FG can be obtained at the same time, minimizing patient burden. Repeat testing with more than a single test is in accordance with national recommendations for the diagnosis of diabetes in asymptomatic patients [3, 48]. These recommend repeat testing for all patients with an abnormal initial test, except for those in a hyperglycemic crisis or classic symptoms of hyperglycemia and a random plasma glucose ≥200 mg/dL. They also recommend that it is preferable that the same test be repeated (at a later date) for confirmation. The value of repeat testing at two points in time on the same patients has not yet been studied in patients taking antipsychotics. In our opinion, all patients taking antipsychotics should be routinely tested for diabetes and prediabetes due to the high risk and adverse consequences. Currently, recommendations for routine testing for diabetes in asymptomatic, undiagnosed adults include adults of any age with BMI ≥25 kg/m^2^ and one or more of the known risk factors for diabetes [53]. We suggest that patients with SMI taking antipsychotic medication is added to the list of known diabetic risk factors.

It is important that once diabetes is detected, that timely and appropriate treatment is given. In the Clinical Antipsychotic Trials of Intervention Effectiveness (CATIE) study, 38% of those with detected diabetes at baseline were left untreated [62]. Mitchell et al (2010) reviewed eleven studies that compared the quality of diabetes care in patients with and without mental illness in routine clinical settings and found significant disparities [63]. Mai et al (2012) studied quality of diabetes care in 139,208 people with mental illness and 294,180 matched controls from Western Australia [64]. Patients had lower rates of screenings (HbA1c, blood lipids), but increased risks of hospitalization for diabetes complications including diabetes-related mortality.

However, it is important to note some considerations when interpreting our results. We did not have prospective data on how pre-diabetes or diabetes might change in this sample. We did not have data on repeat testing which could be examined as a possible diagnostic strategy. One important factor is that we did not have data on end organ dysfunction which is an important and adverse outcome from diabetes. Future research should seek to ascertain this information.

In conclusion, patients with SMI taking antipsychotics are at significantly increased risk of diabetes and, therefore, clinicians must be vigilant for symptoms of diabetes, diabetic risk factors and also screen at regular intervals (we recommend annually). In order to best identify newly redefined diabetes, we recommend a simple biochemical algorithm as follows: step 1. HBA1c ≥5.7%; if negative, test again in 6 months, but if step 1 is positive, proceed to conventional testing (HBA1c and fasting glucose and OGTT at conventional cuts-offs). Patients with diabetes should be referred to an appropriate specialist and at the same time have a review by mental health specialist to clarify which risk factors, including prescription of potentially hazardous antipsychotic medication can be addressed. Such recommendations may be improved by improved integrated and collaborative care between physical and mental healthcare facilities.

## Supporting information

S1 File Full data set. (XLS)
